# Reliability studies of vanadium redox flow batteries: upper limit voltage effect†

**DOI:** 10.1039/d4ra04713c

**Published:** 2024-10-28

**Authors:** Rajankumar Patel, Qian Huang, Bin Li, Alasdair Crawford, Bhuvaneswari M. Sivakumar, Chaojie Song, Zhengming Jiang, Alison Platt, Khalid Fatih, David Reed

**Affiliations:** a Battery Materials & Systems Group, Pacific Northwest National Laboratory Richland WA 99352 USA Qian.Huang@pnnl.gov; b Clean Energy Innovation, National Research Council Canada Vancouver BC V6T 1W5 Canada

## Abstract

All-vanadium redox flow batteries (VRFBs) show promise as a long-duration energy storage (LDES) technology in grid applications. However, the continual performance fading over time poses a significant obstacle for VRFBs. This study systematically investigates the impact of increased upper limit voltage (1.6 V, 1.7 V, and 1.8 V) in the reliability and degradation of a scaled VRFB cell (49 cm^2^) over long-term testing (500^+^ cycles). The findings indicate that higher upper voltages significantly decrease capacity and voltage efficiencies. Although electrolyte remixing can restore the majority of the capacity, it only partially recovers voltage efficiency at 1.7 V and 1.8 V, suggesting substantial cell degradation. Analysis reveals that the overpotential increase induced degradation is mainly contributed by the anode during charging and the cathode during discharging. Increased upper voltage amplifies degradation, with the anode being more affected. As confirmed by electrochemical impedance spectroscopy (EIS) and polarization curves, elevated voltages lead to significant resistance increases, driven by charge transfer resistance (mostly from the anode). Moreover, the morphological, surficial, and electrochemical characterization results of cycled electrodes suggest that the degree and mode of degradation were contingent upon the cutoff voltage. For instance, the cathode experienced severe surface degradation at the maximal upper voltage of 1.8 V. This work highlights the importance of optimizing voltage limits to improve the lifetime of VRFBs and offers valuable insights into the development of predictive models through using accelerated stressor lifetime testing (ASLT) protocols for VRFBs.

## Abstract 2

This study systematically investigates the impact of increased upper limit voltage in the reliability and degradation of a scaled vanadium redox flow battery over long-term testing (500 cycles).

## Graphical abstract

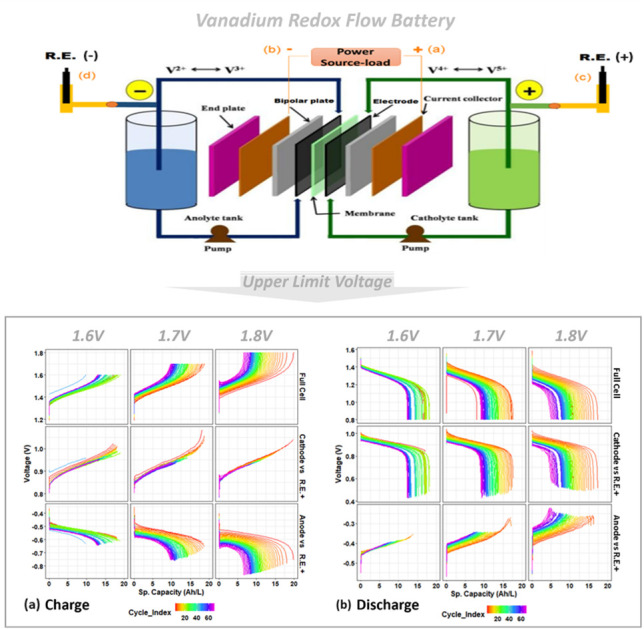

## 1. Introduction

Redox flow batteries have been recognized as a promising stationary energy storage system (ESS) for medium- to long-duration application (4 hours or more) due to their unique features: the separation of energy capacity and power output, high safety, long cycle life, and ease of manufacturing when compared to other rechargeable batteries.^1–3^ Particularly, all-vanadium redox flow batteries (VRFBs), which utilize four oxidation states of vanadium ions to form two soluble redox couples (VO^2+^/VO_2_^+^ and V^2+^/V^3+^) as catholyte and anolyte (see eqn (1)–(3) of electrode and cell reactions), are the most mature redox flow technologies due to their high electrochemical reversibility and high efficiencies.^4^

1 Cathode: VO^2+^ + H_2_O ↔ VO_2_^+^ + 2H^+^ + e^−^, *E*° = 1.00 V

2 Anode: V^3+^ + e^−^ ↔ V^2+^, *E*° = −0.25 V

3 Full Cell: VO^2+^ + V^3+^ + H_2_O ↔ VO_2_^+^ + V^2+^ + 2H^+^, *E*° = 1.25 V

Nevertheless, VRFB technology is plagued by one major technical challenge: the significant capacity decay that occurs during long-term cycling. This is linked to the complex degradation mechanisms that occur within the VRFB. These mechanisms include the following: (i) electrolyte crossover,^4,5^ (ii) electrolyte precipitation (as a strong function of temperature with different valences based vanadium ions),^6^ (iii) oxidation of carbon-based electrodes (typically caused by electrolyte components or high potential),^7,8^ (iv) membrane degradation (mechanically or chemically),^9–12^ and (v) potential degradation from other inactive components (*e.g.* bipolar plate, gasket, and current collector).^13,14^ It is critical to understand the reliability and degradation mechanisms of VRFBs with various cell components at diverse operation conditions.^15^

The traditional RFB performance is assessed in real-time within a cell or stack, a process that is both time-consuming and expensive. Therefore, it is essential to develop accelerated testing methodologies and protocols for a VRFB. Industry has a strong preference for the accelerated stressor lifetime testing (ASLT) protocol, which is highly sought after due to its ability to reduce both time and cost. The fundamental principle of ASLT is that the degradation of VRFB can be accelerated by meticulously selecting the appropriate stressors and their intensity levels. Afterward, these rapid tests can be linked to real-life situations. So far, the development of standardized accelerated testing protocols has been significantly lacking.

Over the past few years, the ASLT has been initiated and methodically studied in our lab. In particular, our previous research has screened and selected stressors, such as high voltage, current density, flow rate, and temperature.^16^ High upper voltage has demonstrated one of the primary stressors that accelerate the degradation of VRFB cells and components.^16,17^ VRFB typically operates with upper voltage limits of 1.55–1.65 V. Higher upper voltage (>1.6 V), which is associated with a higher SOC cutting off (>80%), will potentially lead to side reactions on electrodes, such as the oxidation of carbon electrode on the cathode and the hydrogen evolution reaction on the anode. These side reactions will result in the electrolyte imbalance and/or electrode degradation induced cell performance loss.^17^ Initial research in this area was conducted in a small cell (with an active area of 10 cm^2^ or less) for short-term cycling (50 cycles). The practical implementation of grid-level long duration ESS, on the other hand, necessitates further investigation into a scaled cell and long-term cycling, both of which are currently underexplored. In addition, the degradation mechanism of the cell and its individual electrodes may vary depending on the cell's size, design, component, and testing conditions.^18^

This study systematically investigated the impact of increased upper limit voltage (1.6 V, 1.7 V, and 1.8 V) in the reliability and degradation of VRFBs. ASLT protocols were developed to assess the impact of high voltage. A scaled VRFB cell (49 cm^2^) was subjected to long-term testing (500^+^ cycles). The studies indicate that higher upper voltage limits significantly accelerate cell degradation. The primary degradation mechanisms for whole cell and individual electrodes were identified through a combination of electrochemical analyses and characterizations. This work highlights the importance of optimizing voltage limits to improve the lifetime of VRFBs and provides valuable insights into the development of predictive models by utilizing ASLT protocols for VRFB.

## 2. Experimental

### 2.1. Cell fabrication

**Fig. 1:**
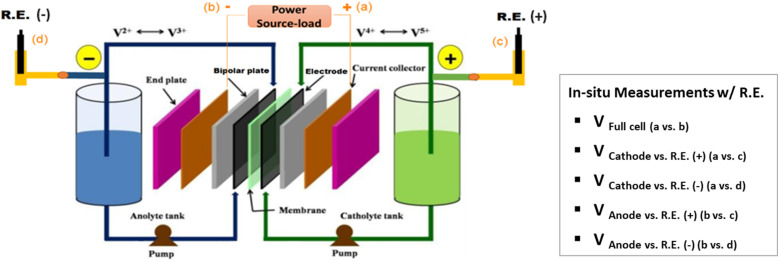
*In situ* measurement setup of a vanadium redox flow battery with the reference electrodes (R.E.) in the inlet tubing of the catholyte and anolyte respectively.

A VRFB (49 cm^2^ active area, Standard Energy Co.) was fabricated by applying a pressure of 0.5 MPa to a stack consisting of a manifold frame, a current collector plate, a graphite bipolar plate (SIGRACET TF6, with a 0.025-inch thickness and flow-through type in no-flow pattern, SGL Group), a bipolar plate gasket, an internal flow frame (3 mm for thickness), a membrane gasket, a graphite felt electrode (GFD 4.6, SGL Group, 7 cm × 7 cm for active area), and a Nafion membrane (N212, Ion Power), for each half-cell (in order from exterior to interior). Prior to cell assembly, the graphite electrodes were thermally treated at 400 °C in air for 6 hours to increase its hydrophilicity. In addition, the two reference electrodes (REs) of Ag/AgCl (with filling solution of 4 M KCl in AgCl, Pine Research Instrumentation) were placed in the inlet tubing of the catholyte and anolyte respectively, as shown in Fig. 1. The R.E.+ and R.E.− stand for the reference electrode in the catholyte and anolyte respectively.

The vanadium electrolytes of 1.6 M V (V^3+^/V^4+^ (50/50) in 2 M H_2_SO_4_ and 0.05 M H_3_PO_4_, GfE)^19^ were used as received. The electrolytes, with the volume of 100 mL per tank, were pumped from the electrolyte reservoirs (Pyrex graduated cylinders) to the flow cell compartments by using a peristaltic pump (Cole-Parmer, Masterflex L/S 7551) at a flow rate of 80 mL min^−1^ through Viton tubing. All the flow battery set was placed inside of a nitrogen gas purged glove bag for testing.

### 2.2. Procedure for baseline testing and measurement

The assembled flow cell was cycled in a charge–discharge process at room temperature with a voltage window between 1.6–0.8 V at a constant current density of 80 mA cm^−2^ using an Arbin cycler. Since the beginning electrolyte solution contains vanadium ions with a valence of 3.5 (a mixture of V^3+^/V^4+^, 50/50) for both catholyte and anolyte, the preparation of V^4+^ for catholyte and V^3+^ for anolyte was achieved by the electrochemical approach in the initial charging process of the cell.^18^

Moreover, the baseline testing procedure involves the electrolyte remixing at every ∼100 cycles, combined with electrochemical impedance spectroscopy (EIS) and polarization curves measurement at the top of charge (TOC) and sampling at the bottom of discharge (BOD) at every ∼50 cycles, until the end of total ∼500 cycles. The cell EIS measurement was conducted using the Gamry instrument with a frequency range of 1 Hz to 100 kHz and a perturbation voltage of 10 mV. Then the EIS curves were analyzed using the Zview program (Scribner). Polarization curves were measured in a charged cell (charged to 1.6 V and then rested for 5 minutes, with an OCV of 1.45–1.5 V). E/i-measurements were carried out from 1.45 V to 0.75 V by reducing the potential every 40 or 30 s by 0.05 V (with 5 s′ rest after each potential measurement). The collected data points of current (i) were the value measured over the last few seconds of each potential step.^18^

### 2.3. Procedure for upper limit voltage stressor testing

The high voltage investigation entails establishing the upper limit voltage at 1.6 V (baseline), 1.7 V, and 1.8 V for each charge cycle, respectively. All cell testing conditions, operation parameters, and measurements are identical to those of baseline testing (refer to “2.2. Procedure for baseline testing and measurement”), with the exception of the upper limit voltage.

### 2.4. Post characterizations

After testing, the cells were disassembled, and samples of the cycled electrodes (cathode and anode) were collected for analysis. The carbon felt (CF) electrodes underwent morphological analysis using scanning electron microscopy (SEM), surface compositional analysis with X-ray photoelectron spectroscopy (XPS), Raman spectroscopy, and electrochemical analysis *via* cyclic voltammetry (CV). Notably, the cycled electrode samples used for SEM were rinsed with deionized (D.I.) water to remove residual electrolyte, ensuring the accurate observation of the CF morphology. However, the electrode samples for XPS and Raman were not rinsed to avoid any potential alteration or damage to the CF surface functionality that could result from the D.I. water rinsing.

The morphology of the electrodes was studied by JSM-IT2000 scanning electron microscopy. The JEOL Backscatter Electron Detector is optimized for a 10 mm working distance for Energy Dispersive Spectra. XPS measurements were performed by Nexsa Thermo Fisher Scientific spectrometer, using a focused Al Kα monochromatic X-ray source (1486.6 eV) operated at 72 W and a high-resolution spherical mirror analyzer with a 50 eV pass energy. The data acquisition was carried by a 300 μm diameter X-ray beam and the emitted photoelectrons were collected at the analyzer entrance slit normal to the sample surface. The chamber pressure was maintained at ∼5 × 10^−9^ Torr during the measurements. All the XPS peaks were charge referenced with C 1s binding energy at 284.8 eV. XPS data was analyzed by Casa XPS software using Shirley background correction. The Raman spectroscopy was performed using in-house spectrometer equipped with 633 nm laser and a CCD detection system (Andor, Shamrock 303i and iDus 416). The Raman data shown is spatially (100 spectra in a 10 × 10 μm^2^ area) and temporally (integration time of 300 s) averaged to ensure sample integrity throughout the measurements. The detailed CV experimental procedure is provided in the ESI.†

## 3. Results and discussion

### 3.1. Cycling performance

**Fig. 2:**
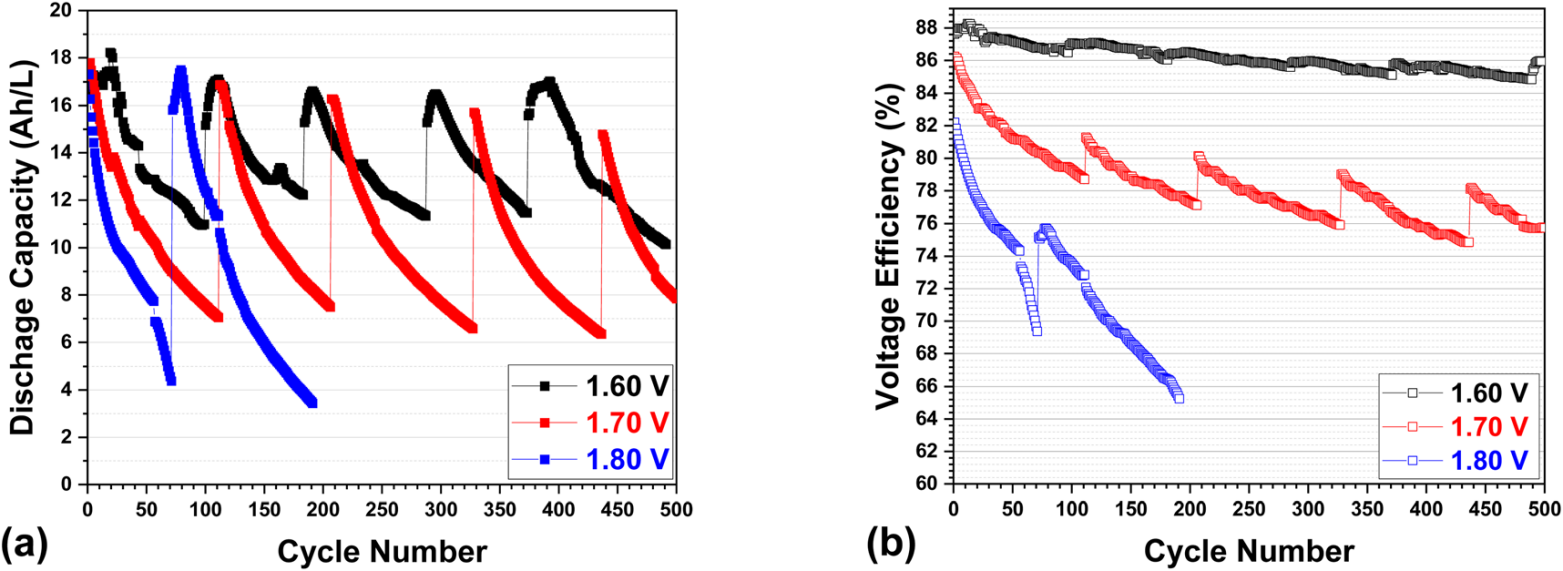
(a) Specific discharge capacity (A h L^−1^) and (b) voltage efficiency (%) of a VRFB as a function of cycle number at different upper limit voltages of 1.6, 1.7, and 1.8 V.

An in-depth investigation of the specific discharge capacity and voltage efficiency of VRFBs with different upper voltage cutoff values (1.6, 1.7, and 1.8 V) is illustrated in Fig. 2. The baseline testing, represented by the black curve at an upper voltage of 1.6 V in Fig. 2a, demonstrates a substantial drop in capacity, maintaining a 65% of its discharge capacity after 99 cycles. This considerable capacity loss is primarily attributable to electrolyte crossover during real charge–discharge cycling, which involves a change in the concentration and volume of active materials (vanadium ions with varying valences) in each catholyte and anolyte. This crossover phenomenon was previously systematically investigated by our group.^4,18^ As stated in the experimental section, the cell testing procedure involves the electrolyte to be remixed approximately every 100 cycles. It has been observed that this remixing process mostly restores the whole capacity of the cell. A further analysis of the baseline testing in the voltage efficiency demonstrates a remarkable retention of around 98.7% after 99 cycles (from 87.6% to 86.5% of VE), as illustrated in Fig. 2b. However, the electrolyte remixing procedure cannot fully restore the VE. The gradually decreasing VE over 500 cycles (with electrolyte remixing occurring every 100 cycles) indicates that the carbon felt-based electrode degrades incrementally during long-term cycling.

It is worth noting that an increase in upper voltage results in a more pronounced decrease in both discharge capacity and voltage efficiencies. After approximately 100 cycles (before electrolyte remixing), the discharge capacity retention decreased from 65% for 1.6 V (baseline) to 44% for 1.7 V, and further to 25% for 1.8 V (after 70 cycles). Meanwhile, the VE retention decreased moderately from 98.7% for 1.6 V to 92.0% for 1.7 V (after approximately 100 cycles), and more significantly to 84.4% for 1.8 V (after 70 cycles). Similar to in the baseline testing, electrolyte remixing can restore the majority of the capacity, but it can only restore a portion of the VE when the upper voltage is raised (up to 1.7 or 1.8 V); this suggests that a higher upper voltage may cause more significant cell degradation not only from electrolyte (crossover) but electrode as well.^17^

Notably, 1.6 V and 1.7 V cells can withstand more than 500 cycles, with electrolyte remixing (every 100 cycles) restoring capacity for the most part. However, the 1.8 V cell is incapable of enduring 200 cycles despite the electrolyte remixing process, as evidenced by the precipitous decrease in VE, which indicates that the maximum upper voltage of 1.8 V has a major and irreversible impact on cell (electrode) degradation.

### 3.2. Charge–discharge profiles

**Fig. 3:**
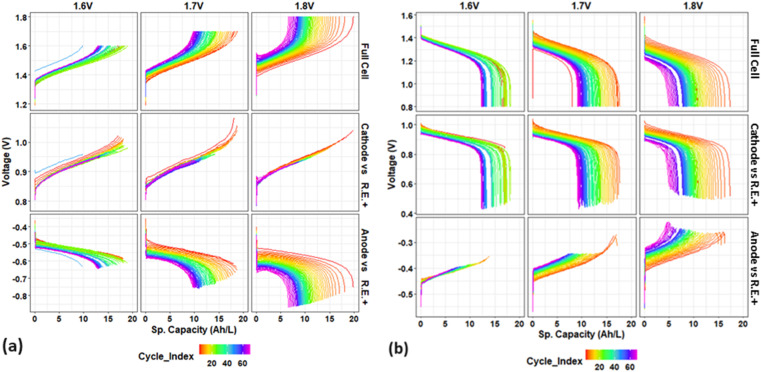
Voltage profiles of full cell and its individual electrodes (cathode or anode *vs.* RE+) *vs.* specific capacity (A h L^−1^) for initial 70 cycles: during (a) charge and (b) discharge process, at different upper limit voltages of 1.6 V, 1.7 V, and 1.8 V.

Moreover, Fig. 3 displays the voltage profiles of the full cell and its individual electrodes in relation to their specific capacities over the initial 70 cycles (before electrolyte remixing of all cells). The profiles comprise (a) charge and (b) discharge processes, with upper voltages set at 1.6 V, 1.7 V, and 1.8 V.

Overall, as cycling progresses, the voltage profiles for each full cell shift positively during charge (Fig. 3a) and negatively during discharge (Fig. 3b), indicating an increase in overpotential that leads to capacity fading and cell degradation. The voltage profile shifting trend of full cell becomes more pronounced at higher upper voltages. At an elevated upper voltage (1.6 V, 1.7 V and 1.8 V), the voltage curve shifts more significantly towards a higher and shorter level during charge and to a lower and shorter level during discharge. Consequently, the overpotential increases by 0.04/0.06 V, 0.1/0.15 V, and 0.15/0.22 V (charge/discharge) for 1.6 V, 1.7 V, and 1.8 V over the initial 70 cycles.

As previously stated, reference electrodes were positioned within the inlet tubing of the catholyte (+) or anolyte (−) in this study, which enables the *in situ* monitoring individual electrode voltage over long-term cycling. The individual electrode voltage profiles (cathode or anode *vs.* R.E.+) that correspond to the full cell profiles are also displayed in Fig. 3. The cathode and anode voltage profiles (*vs.* R.E.+) exhibit distinct behaviors and contribute differently to the full cell degradation during cycling.

During the charge process (Fig. 3a), the cathode voltage curves remain relatively stable, whereas the anode voltage curves decrease significantly by ∼0.03, 0.09, and 0.15 V for 1.6, 1.7, and 1.8 V, respectively, over the first 70 cycles. The alignment between the voltage trends of anode and full cell suggests that the anode is primarily responsible for the observed overpotential increase during charging.

During the discharge process (Fig. 3b), the voltage curves of full cells decrease by 0.06, 0.15, and 0.22 for the first 70 cycles, with the cathode curve decreasing significantly by 0.05 V, 0.1 V, and 0.14 V for 1.6, 1.7, and 1.8 V, respectively. The anode voltage curves show a negligible shift for 1.6 V but increase more significantly by 0.05 V and 0.08 V for 1.7 V and 1.8 V, respectively. This indicates that while the cathode predominantly contributes to performance degradation during discharge, the anode's contribution becomes more significant at higher upper cut-off voltages over cycling.

It should be noted that the anode degradation is associated with the V(ii) adsorption on the carbon electrode surface, which deteriorates the V(ii)/V(iii) reaction, as reported previously.^20^ Our work's observation that the anode contributes more to overpotential during charge is in good agreement with the V(ii) adsorption phenomenon. During charge, adsorption of V(ii) on the electrode surface inhibits the anode reaction where V(iii) is supposed to move to the electrode surface and then be reduced to V(ii). This V(ii) adsorption induced the degradation results in a higher overpotential on the anode side. However, during discharge, the opposite reaction on the anode might happen easier. Despite of the same phenomenon of V(ii) adsorption, the absorbed V(ii) on the electrode surface might be easily oxidized, resulting in a lower overpotential. In general, the V(ii) adsorption induced anode kinetics degradation might dominate the overpotential increase during charge, while both anode and cathode contributed to the overpotential during discharge. In addition, the anode voltage (*vs.* R.E.+) may include membrane effect (due to the position of R.E.+ in the inlet tubing of the catholyte side), but the influence of the membrane on cell degradation upon cycling is probably insignificant when comparing our testing results employing REs at various positions.

The results indicate that the overpotential contributions from the cathode and anode can vary during the charge and discharge processes. This is due to the fact that overpotential in a VRFB is a complex phenomenon that is influenced by a variety of factors, such as reaction kinetics, internal resistance, and mass transport, which are associated with each component of the cell (electrode, electrolyte, membrane, *etc.*). Therefore, the diverse degradation behavior of individual electrodes can be a result of the opposing electrochemical reactions that occur during charging and discharging.

In summary, the anode primarily contributes to the overpotential-induced cell degradation during charging, while the cathode is the primary contributor during discharging, which is in a good agreement with our prior research.^18^ This degradation trend associated with the contribution of each individual electrode during charging and discharging becomes more pronounced as the upper limit voltage rises; further, the anode's contribution during discharging increases. Overall, as the upper voltage increases, the overpotential induced degradation of both electrodes becomes more pronounced, although it is comparatively more pronounced on the anode side.

### 3.3. OCV and overpotentials at the top of charge and bottom of discharge

**Fig. 4:**
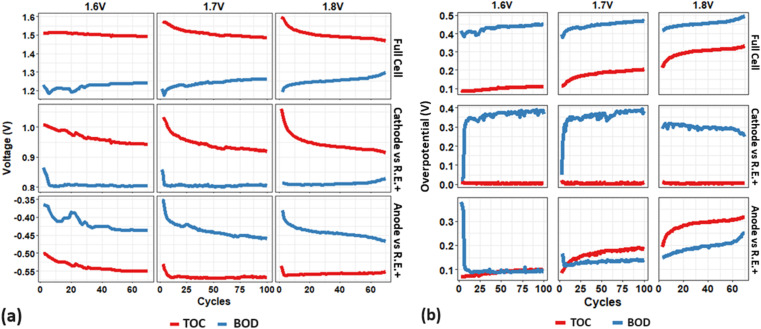
(a) OCV and (b) overpotentials at the top of charge (TOC) and bottom of discharge (BOD) as a function of cycle numbers for the full cell and individual electrode (cathode *vs.* RE+), for initial 100 cycles or 70 cycles at different upper limit voltages of 1.6 V, 1.7 V, and 1.8 V.

Fig. 4 presents (a) the open-circuit voltage (OCV) and (b) the overpotential at the top of charge (TOC) and bottom of discharge (BOD) as a function of cycle numbers for the full cell and individual electrodes. As a key indicator of the state of charge (SOC) in a VRFB, the OCV is directly related to the concentration of active materials (vanadium ions) in the catholyte and anolyte.^21^

The OCV (Fig. 4a) shows approximately 1.5 V at the TOC (equivalent to approx. 78% SOC^21^) during the initial 70 cycles of a 1.6 V (baseline) cell, where it ranges from 1.18 to 1.25 V at the BOD. The increase in upper voltage to 1.7 V and 1.8 V results in a corresponding rise in the OCV at the TOC to 1.56 V and 1.6 V, respectively. These OCV values correspond to a higher SOC of 90% and ∼100%, respectively.^22^ The cathode side demonstrates more contribution than the anode side, as evidenced by the cathode's significantly increased OCV (1.0 V, 1.03 V, and 1.06 V) and the anode's marginally decreased OCV (−0.50, −0.53, and −0.54 V) in response to the elevated upper voltage (1.6, 1.7, 1.8 V). The OCV at the TOC for 1.7 and 1.8 V, however, decreases substantially and stabilizes at approximately 1.5 V (near the value in the baseline cell) after 20 cycles. The OCV curves at the BOD remain consistent across the three different cut-off voltages, displaying a similar trend for individual electrodes. This consistency suggests that higher upper cut-off voltages result in a higher OCV/SOC at the TOC in the initial cycles, with negligible changes at the BOD.

Then increased degradation due to higher upper voltages (1.7 V and 1.8 V) leads to a faster drop in the OCV at the TOC, which stabilizes after the first 20 cycles. Notably, after about 60 cycles, the OCV at the TOC starts to decrease below the baseline value, indicating cell degradation driven by higher upper voltages. This degradation is reflected by the decrease in upper OCV/SOC (red curve) and the increase in lower OCV/SOC (blue curve), particularly after 60 cycles, as shown by the 1.8 V full cell OCV curves. This is probably related to electrolyte degradation (SOC) and reduced capacity, which is primarily caused by the cathode side.

In general, the overpotentials (Fig. 4b) of the 1.6 V baseline full cell at the BOD (400–450 mV) are approx. three times higher than those at the TOC (100 mV), mostly dominated by the cathode. The overpotentials of the cathode at the BOD (350–400 mV) align well with those of the full cell and are much higher than at the TOC (<50 mV), whereas the anode overpotentials remain stable and relatively low (50–100 mV) at both the BOD and TOC, except for the initial 10 cycles. With an increased upper voltage up to 1.8 V, the overpotential at the BOD (blue curves) does not change significantly initially but increases more noticeably (up to 500 mV) after 60 cycles, predominantly due to the anode. On the other hand, the increase in upper voltage has a more significant impact on the overpotential increase at the TOC: 100 mV, 130–200 mV, and 220–350 mV for upper cut-off voltage of 1.6, 1.7, and 1.8 V, which is also mainly contributed by the anode.

The analysis of Fig. 4 reveals that increasing the upper cut-off voltage in a VRFB leads to an increase in the OCV (SOC) at the TOC during the initial cycles, primarily contributed by the cathode. However, further degradation over cycling results in the OCV (at the TOC) dropping significantly and approaching a minimum value (that is more evident in a 1.8 V cell). In contrast, the OCV at the BOD remains relatively stable across different voltages. Higher upper voltages result in a noticeable increase in overpotentials, most notably at the TOC, where the impact is more significant and primarily attributed to the anode. These findings emphasize the important role of both the cathode and anode in cell performance and degradation. Specifically, the cathode primarily affects the initial SOC and total overpotential, while the anode is primarily responsible for long-term increases in overpotential. It is well known that overpotential (at the TOC and BOD) play a significant role in determining the charge or discharge capacity of a VRFB. Thus, our results indicate that as the upper voltage increases, the cathode is more responsible for determining the cell's capacity (which is decreasing), whereas the anode contributes more to the cell's degradation (which is more significant).

### 3.4. Cell resistance characterizations

**Fig. 5:**
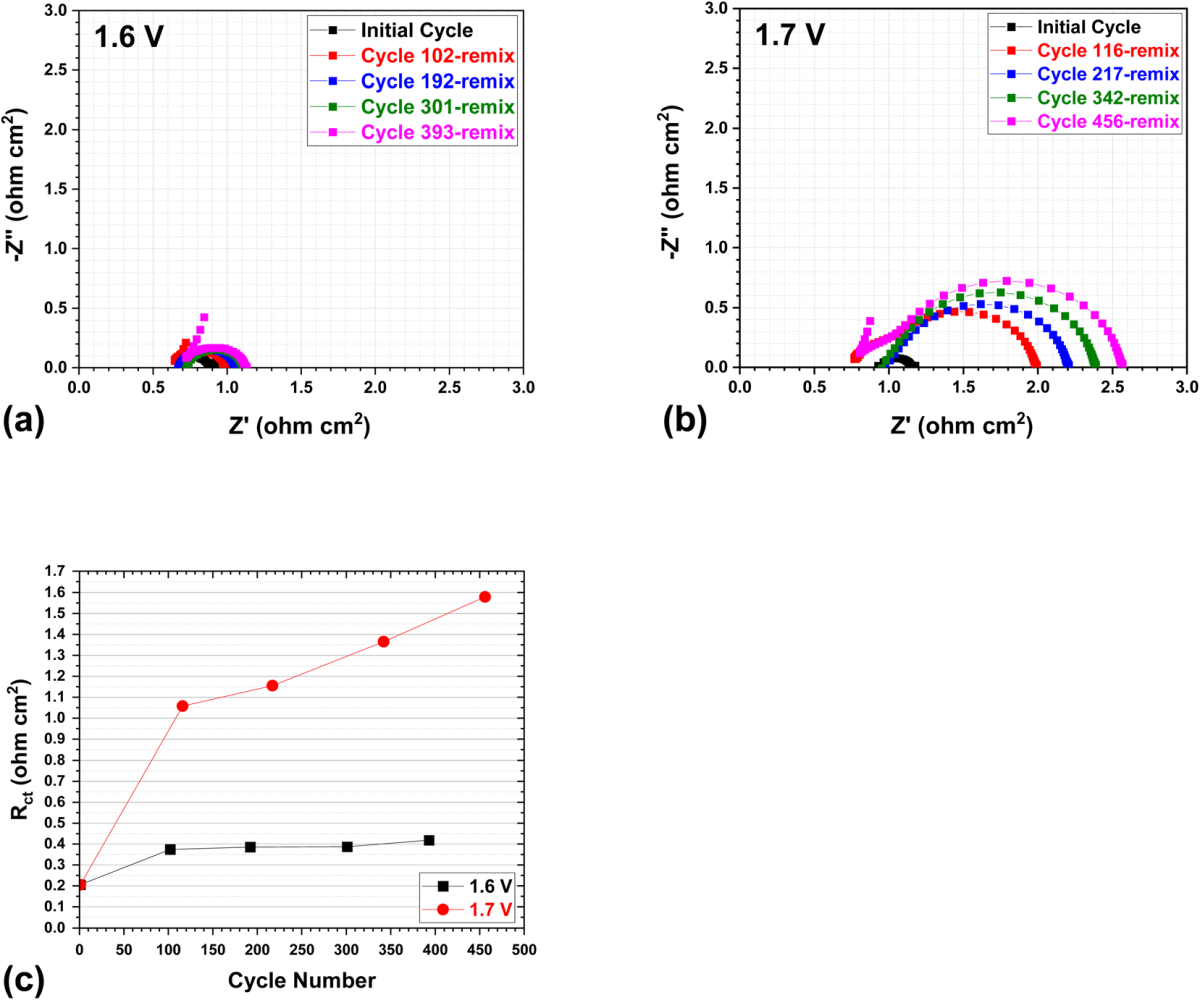
Typical Nyquist plots at the top of charge (TOC) after remixing (at every ∼100 cycles), with an upper cut-off voltage of (a) 1.6 V and (b) 1.7 V; and (c) the charge transfer resistances (*R*_ct_) of the 1.6 V and 1.7 V cells by EIS curves (5a and 5b) analysis using the Zview program (Scribner).

To track the change in cell resistance during long-term cycling, the EIS was measured for cells at the top of charge (TOC) over a period of cycles. Fig. 5 depicts the typical Nyquist plots for cells at initial cycles and after each electrolyte remixing process, with upper voltages of 1.6 V and 1.7 V. The ohmic resistance of the cell is denoted by the *x*-intercept, whereas the charge transfer resistance (*R*_ct_) is represented by the semicircle.^23,24^ The resistance values for each Nyquist curve are calculated and separated using the Zview Program (Scribner). The ohmic resistance remains constant for each cell throughout long-term cycling, whereas the charge transfer resistance increases gradually with cycling. Note that these EIS measurement was carried out after each electrolyte remixing of the cell so as to exclude the electrolyte crossover induced cell degradation. Specifically, the charge transfer resistance (represented by the semicircles of the Nyquist curves) exhibits a slight increase for the 1.6 V cell during cycling, while it demonstrates a more substantial rise for the 1.7 V cell, as demonstrated by Fig. 5c. As a consequence, an elevation in upper voltage leads to a significant surge in resistance throughout the cycling process, which corresponds favorably with the increase in overpotential as discussed earlier. This increase primarily stems from the heightened charge transfer resistances, which are unrecoverable through electrolyte remixing and are likely caused by the electrode degradation of VRFBs. Further EIS measurement on individual electrode–anode *vs.* R.E.+ in Fig. S1† indicates that for 1.6 V and 1.7 V cells, the anode charge transfer resistance is the primary factor in the total resistance at the TOC, as evidenced by the almost identical charge transfer resistance (semicircle in curve) from the anode (Fig. S1†) *vs.* full cell (Fig. 5). This implies that the performance of these cells may be influenced by the anode reaction and the potential membrane effect, particularly in a charged state. This is in accordance with the overpotential results in Fig. 3, which indicate that the anode exhibits a more pronounced overpotential-induced degradation than the cathode during charging, as a result of the V(ii) adsorption-induced anode kinetics degradation.

In addition, the polarization curves were assessed to determine the performance degradation of the cell at upper voltages of 1.6 V and 1.7 V, respectively, after first electrolyte remixing (refer to Fig. S2†). As the upper voltage is increased, the results indicate that the performance degradation (ohmic loss) of the full cell becomes more pronounced (see ESI†), in good agreement with the EIS results.

### 3.5. Electrode characterizations

**Fig. 6:**
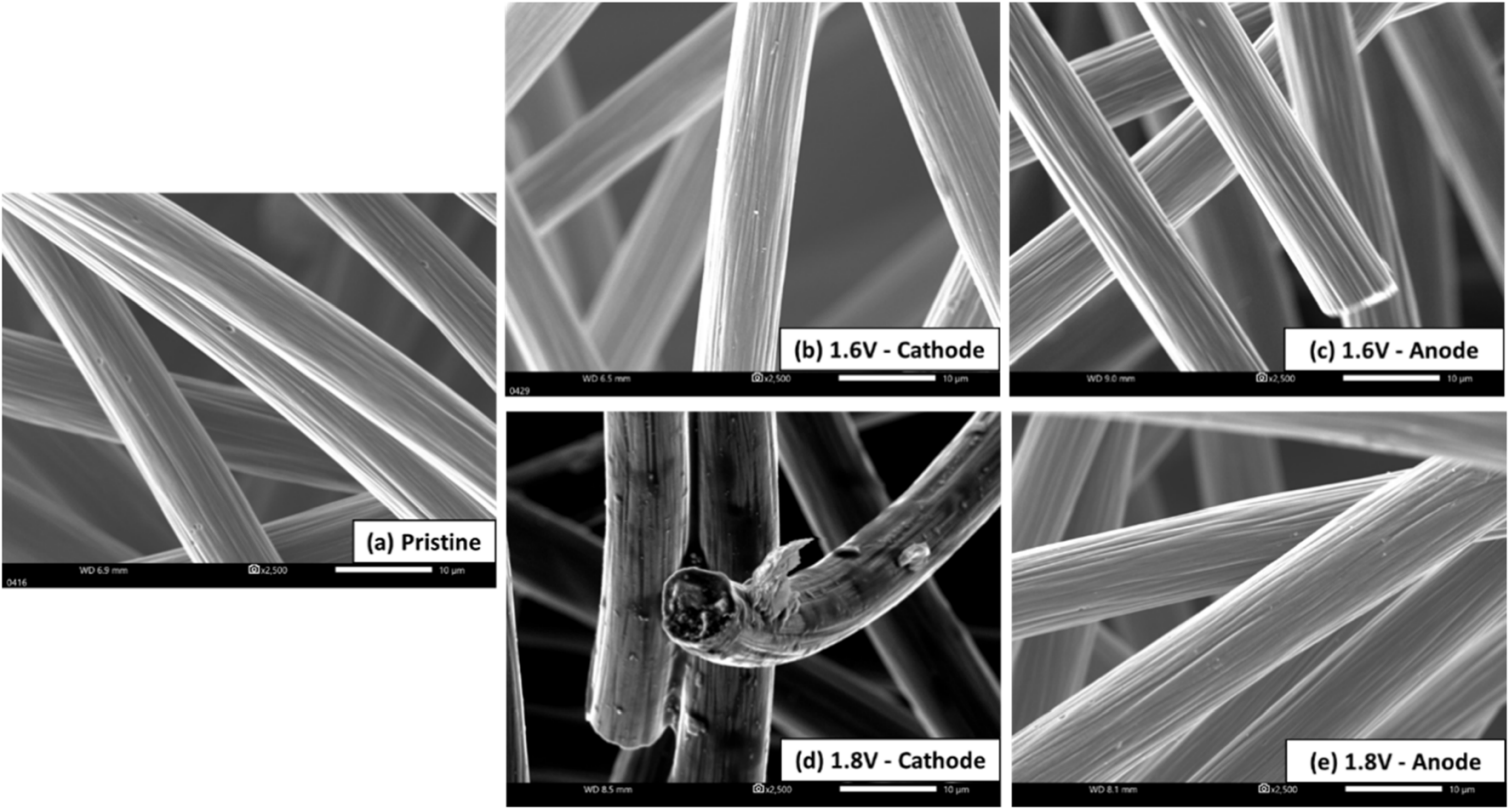
Scanning electron microscopy images of the electrodes: (a) pristine CF, and (b–e) cathode and anode after cycling at different upper cut-off voltage (1.6 or 1.8 V).

To gain a better understanding of the effect of the upper voltage limit on electrode degradation after testing, we conducted additional characterizations of the tested CF electrodes using SEM, XPS, and CV. These techniques were employed to analyze the tested electrodes' morphology (Fig. 6), surface functional groups (Fig. S3 and S4†), and electrochemical properties (Fig. S5†).

Fig. 6a shows the typical edgy fiber morphology of the pristine CFs. After cycling at an upper voltage of 1.6 V, both the cathode and anode CFs (Fig. 6b and c) retained a morphology similar to that of the pristine CFs, indicating that baseline testing under mild conditions for over 500 cycles did not significantly affect electrode morphology. However, increasing the upper voltage to 1.8 V led to noticeable surface deterioration on the cathode CFs, including the appearance of varying hues and possible surface layer peeling (Fig. 6d). In contrast, the anode CFs continued to exhibit characteristics identical to those of the pristine electrodes or 1.6 V CFs (Fig. 6e). The degradation observed at 1.8 V on the cathode CFs likely caused the loss of active surface functional groups, contributing to the elevated overpotentials of the cathode during cycling (especially during discharging) at this voltage, as seen in Fig. 3b. SEM analysis indicates that higher upper voltage limits (1.8 V) result in significant surface degradation of the cathode CFs while cycling under mild operating conditions (1.6 V) over long cycles had no noticeable impact on electrode morphology.

To confirm the impact of the upper limit voltage on electrode degradation, XPS^25,26^ is further augmented to learn surface functional group information of cycled CF electrodes. Fig. S3† shows the high resolution C 1s spectra for the cycled CF electrodes (both cathode and anode) at an upper voltage of 1.6 V or 1.8 V, respectively, along with the corresponding deconvoluted peaks. From these spectra, the ratio of sp2/sp3 was calculated to quantitively compare the effects of different upper voltages on the surface functions of electrode, as shown in Fig. S3,† indicating the cathode of 1.8 V cell is undergoing significant surface function group loss during cycling, which is correlated with more pronounced electrode degradation attributable to the higher upper limit voltage, in good agreement with the SEM results. The surface degradation of the carbon electrode after long-term cycling is further verified by the supplementary Raman characterization (Fig. S4†). The 1.8 V cathode exhibits a higher extent of defects (more surface degradation) as evidenced by the higher ratio of main carbon peak intensity *I*_D_/*I*_G_ (the percentage of sp3 carbon)^27^ in comparison to the 1.8 V anode and 1.6 V electrodes. The results are also consistent with those of the SEM and XPS.

Additionally, the tested electrodes were characterized by CV to assess their electrochemical properties. Specifically, the CF electrodes were analyzed in a pure acid solution (2 M H_2_SO_4_) to evaluate its inherent properties, while its activity was measured in a vanadium solution (0.2 M VOSO_4_ -2 M H_2_SO_4_), as shown in Fig. S5 and Table S1.† Overall, the CV results indicate that the CF electrodes degraded during VRFB testing. However, the degree and mode of degradation were dependent on the cutoff voltage. For the 1.6 V CFs, the anode (1.6 V(−)) exhibited more severe degradation than the cathode, while for the 1.8 V CFs, the cathode degraded more significantly than the anode. Comparatively, the 1.8 V cathode exhibited the most severe degradation, as evidenced by the weakest reduction peak of V(v). These findings align well with the results from cell performance (charge–discharge curves) and other characterizations.

In summary, the degree and mode of cell degradation are notably influenced by the cutoff voltage. As the upper voltage limits increase, the degradation behavior of the individual electrodes-either the cathode or anode-varies. Through comprehensive characterization techniques, including surface analysis *via* XPS and Raman, morphology assessment using SEM, and electrochemical evaluation by CV and EIS, as well as cell testing on individual electrodes, it was generally observed that the anode experiences more severe degradation than the cathode in a 1.6 V cell. Conversely, in a 1.8 V cell, the cathode undergoes more substantial degradation than the anode. The cell degradation at lower upper voltage limits, such as 1.6 V, which is more dominated by the anode, can be primarily attributed to the degradation of anode kinetics due to V(ii) absorption on the anode surface and further anolyte degradation from electrolyte crossover.^4,20^ However, when the upper voltage limit is increased to 1.8 V, the cathode becomes more susceptible to oxidative damage,^17^ leading to significant surface function loss (as indicated by XPS, Raman and CV) and morphological degradation (as shown by SEM). At the higher upper voltage, the anode continues to contribute to a notable increase in overpotential due to V(ii) absorption and electrolyte crossover. But V(ii) absorption also helps inhibit the hydrogen evolution reaction (HER) side reaction^20^ at higher voltages, potentially safeguarding the anode from permanent damage, unlike the cathode. These complex and interconnected effects from individual electrode (cathode/anode) and electrolyte (catholyte/anolyte) collectively contribute to the overall performance degradation of the VRFB. Further studies are necessary to fully elucidate these mechanisms.

## 4. Conclusions

This study investigates the impact of high upper voltage limits (1.6–1.8 V) on the reliability and degradation of a scaled VRFB cell with a 49 cm^2^ active area over 500+ cycles. The findings indicate that higher upper voltage limits (1.6–1.8 V) significantly reduce discharge capacity and voltage efficiency in VRFB cells, with more pronounced degradation at 1.8 V. While electrolyte remixing effectively restores most of the capacity, it only marginally recovers voltage efficiency at higher voltages, indicating irreversible cell degradation. Further analysis reveals that the overpotential increase induced degradation is mainly contributed by anode during charging and cathode during discharging. Increased upper voltage amplifies degradation, with the anode being more affected. As confirmed by EIS and polarization curves, elevated upper voltages result in substantial resistance increases, linked to higher charge transfer resistance, which electrolyte remixing cannot recover. Moreover, the morphological (SEM), surficial (XPS and Raman), and electrochemical (CV) characterizations of cycled electrodes suggest that the degree and mode of degradation were contingent upon the cutoff voltage. Higher upper voltages, particularly at maximum upper voltage of 1.8 V, cause significant surface degradation of cathode carbon felt with reducing active surface functions. This work highlights the significance of optimizing voltage limits to enhance the lifetime of VRFBs and provides valuable insights into the development of predictive models for VRFB lifetime. Our pioneering development and application of ASLT protocols demonstrate the importance of this approach. We will continue to refine ASLT protocols and investigate other potential stressors in the future.

## Data availability

Data are not publicly accessible; however, they may be obtained upon request.

## Conflicts of interest

There are no conflicts to declare.

## Supplementary Material

RA-014-D4RA04713C-s001
