# Supplementary material for: Reliability studies of vanadium redox flow batteries: upper limit voltage effect
Source: RSC Adv. 2024 Oct 28;14(46):34381–9. doi: 10.1039/d4ra04713c (PMC11515847; doi:10.1039/d4ra04713c)
Supplement: RA-014-D4RA04713C-s001 [file RA-014-D4RA04713C-s001.pdf]

Supplementary Information (SI) for RSC Advances.
This journal is © The Royal Society of Chemistry 2024

Supplementary Information

Reliability Studies of Vanadium Redox Flow Batteries: Upper Limit Voltage Effect

Rajankumar Patel [a], Qian Huang [a *], Bin Li [a], Alasdair Crawford [a], Bhuvaneswari M Sivakumar [a], Chaojie Song [b], Zhengming Jiang [b], Alison Platt [b], Khalid Fatih [b], and David Reed [a]

[a] Battery Materials & Systems Group, Pacific Northwest National Laboratory, Richland, WA 99352, USA

[b] Clean Energy Innovation, National Research Council Canada, Vancouver BC V6T 1W5, Canada

[*] Corresponding author Email Address: Qian.Huang@pnnl.gov

Experimental - Cyclic Voltammetry (CV)

Materials

$VOSO_4.xH_2O$ (99.5%) was purchased from Noah Chemicals, USA, H_2SO4 was obtained from Sigma Aldrich.

Procedures

The carbon felts (CF) were characterized by cyclic voltammetry in a three-electrode cell using a Solartron Potentiostat 1287. The working electrode is CF hooked by a gold wire as current collector. Gold mesh was used as counter electrode, and Ag/AgCl was used as reference electrode.

The tested CFs were washed by deionized (D.I.) water to remove the adsorbed vanadium. 0.71 cm^2 of CF was cut from the cathode/anode using a 3/8-hole puncher. The felt was weighed on a microbalance before the CV testing.

The property of CF was characterized in 2 M H_2SO_4 in a potential range of 0 – 1.2 V vs Ag/AgCl, with a scan rate of 10 mV s^{-1}. The activity of carbon felt was measured in 0.2 M $VOSO_4$- 2 M H_2SO_4 in a potential range of 0 – 1.25 V vs Ag/AgCl, with a scan rate of 1 mV s^{-1}. The current of the CV was normalized by the CF weight.

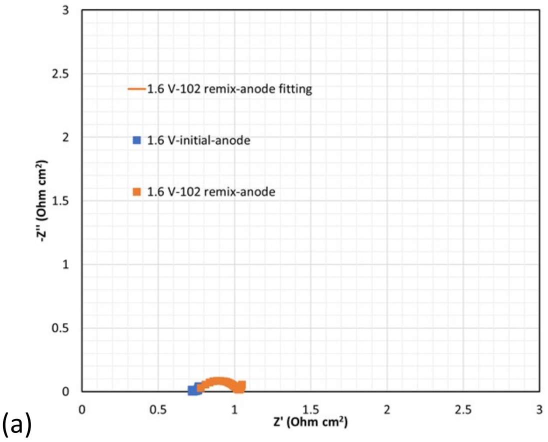

(a)

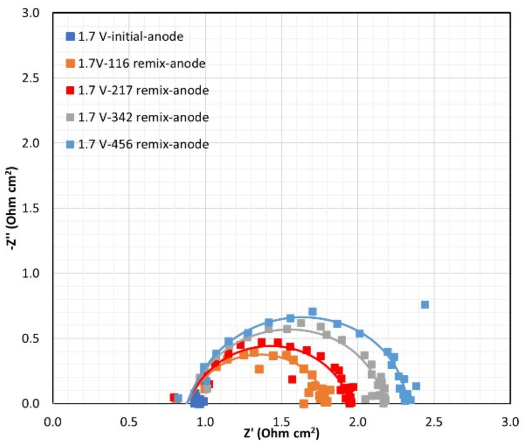

(b)

Figure S1 Typical Nyquist plots of the anode at the top of charge (TOC) after remixing, with an upper cut-off voltage of (a) 1.6 V (at ~100 cycles) and (b) 1.7 V (at each ~100 cycles).

* The cathode's typical Nyquist plots consisted solely of a quasistraight line, devoid of any semicircle curve, which is not depicted.

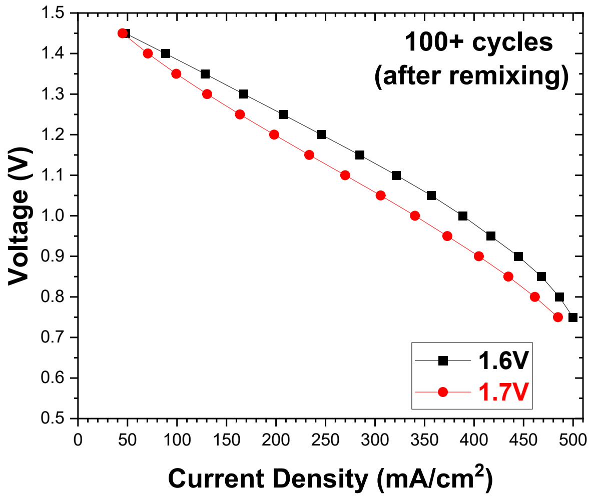

Figure S2 Polarization curves of a vanadium redox flow battery after ~100 cycles (after remixing) at different upper cut-off voltage: 1.6 V (black), and 1.7 V (red).

Through the analysis of polarization curves,[1] the principal losses detected in a vanadium redox flow battery (VRFB) are as follows: i) kinetic activation polarization; ii) ohmic polarization (iR losses); and iii) mass transport limitation. These losses are designated to the three regions in a generalized polarization curve that progress from low to high current density.[2] In contrast to the generalized polarization curve of a VRFB (see Fig. 2 in the published literature 2), each curve in Figure S2 exhibits only two characteristic regions of ohmic loss and transport loss, with no activation loss region. As the upper voltage is increased, the cell performance degradation becomes more pronounced, as indicated by the steepening ohmic loss -PC slopes when the upper voltage was raised to 1.7 V.

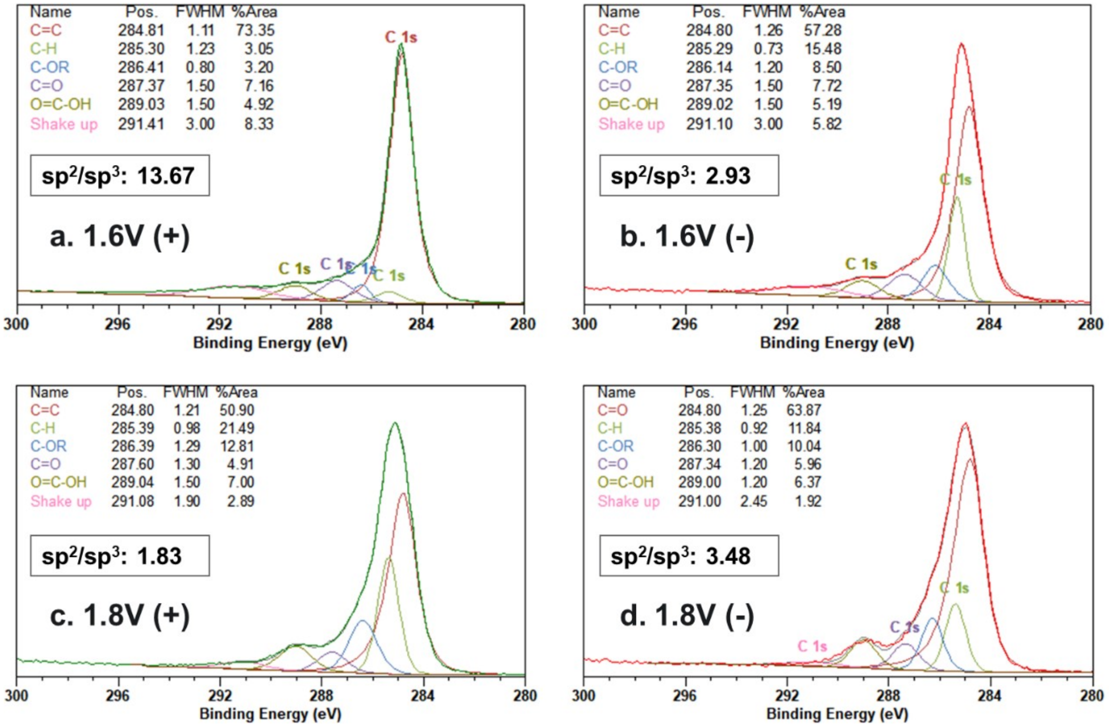

Figure S3 C 1s XP spectra of the electrodes (cathode (+) and anode (-)) after testing at different upper limit voltage: 1.6 V and 1.8 V.

The spectra of high resolution C1s for the cycled CF electrodes (both cathode and anode) at an upper voltage of 1.6 V or 1.8 V, respectively, are depicted in Figure S3 (with the corresponding deconvoluted peaks), by which the ratio of sp2/sp3 (derived from C1s) can be calculated to compare the impacts of varying upper voltages on the electrode surface. The calculated sp2/sp3 ratio exhibits a nearly identical value for the anode in both 1.6 V and 1.8 V cells. However, the corresponding ratio for the cathode in the 1.8 V cell is considerably lower than in the 1.6 V cell. This discrepancy suggests that the cathode is undergoing surface function group loss during cycling, which is correlated with more pronounced electrode degradation attributable to the higher upper limit voltage (1.8 V), in good agreement with the SEM results. Note that the XPS measurement utilizes cycled CF with the remaining electrolyte desiccated, which introduces an additional O source (from SO_4^{2-}). Therefore, the conventional method for determining the ratio of O/C or C=O/C-O (derived from O1s) is unsuitable for this XPS analysis.

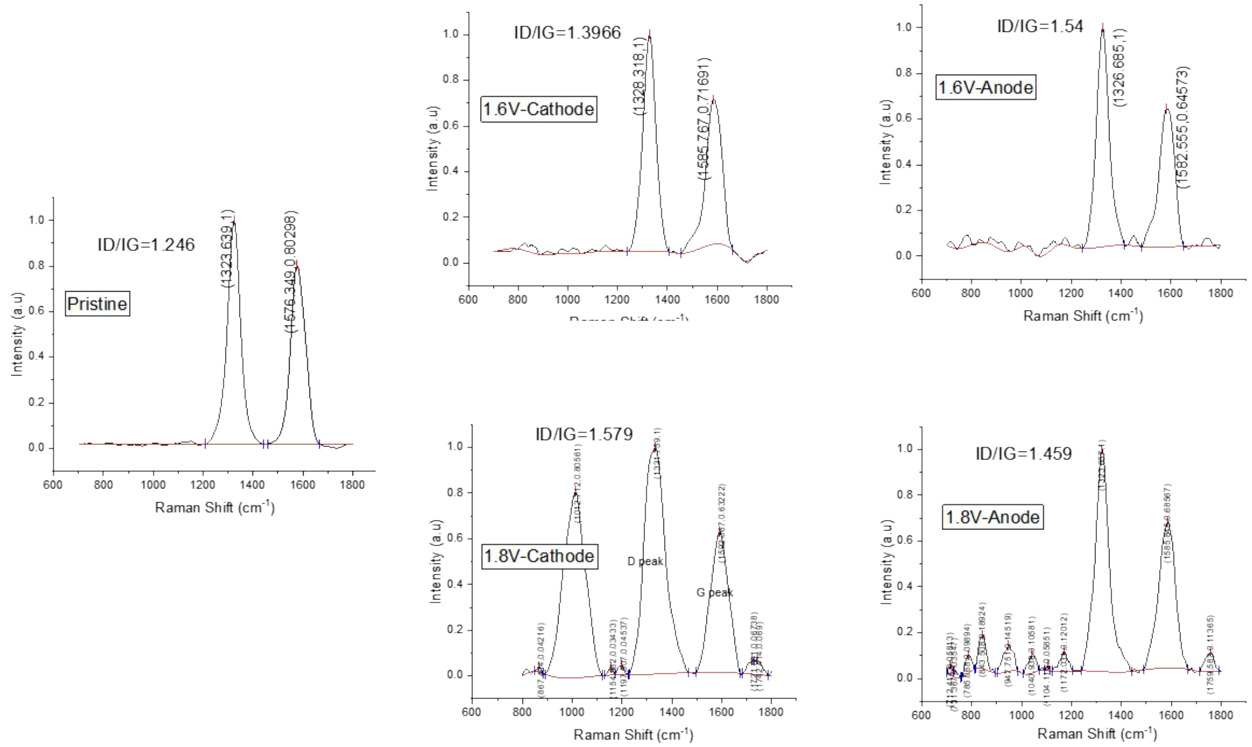

Figure S4 Raman spectra of the electrodes: pristine CF, and cathode and anode after cycling at different upper cut-off voltage (1.6 or 1.8 V).

(a)
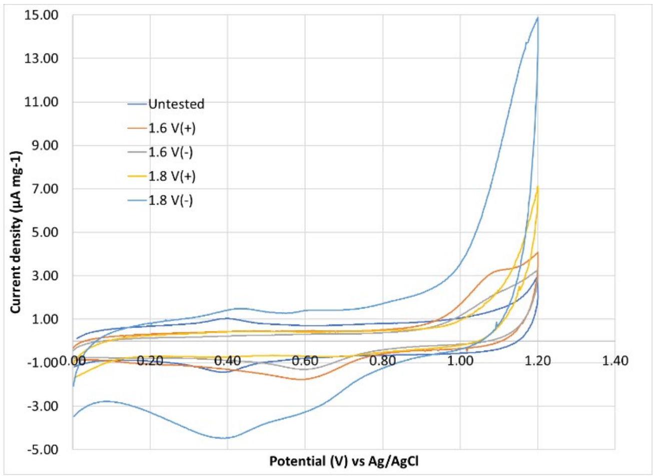

(b)
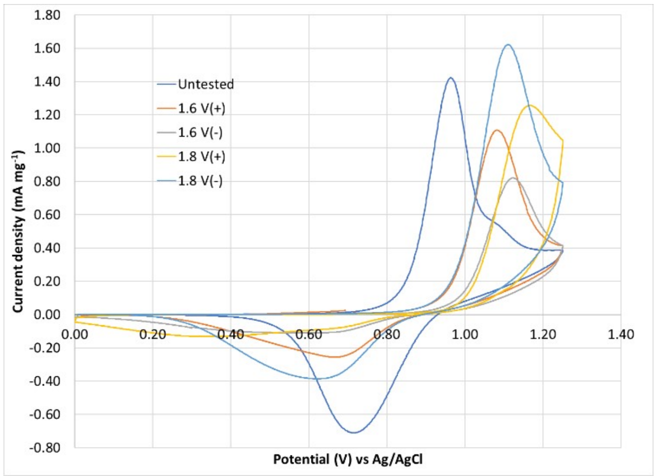

Figure S5 Cyclic voltammogram of caron felts untested, and cathode (+) and anode (-) after cycling at different upper cut-off voltage (1.6 or 1.8 V), in (a) 2 M H_2SO_4. Scan rate: 10 mV/s; and (b) 0.2 M $VOSO_4$ - 2M H_2SO_4, scan rate: 1 mV/s.

Figure S5 (a) compares the CV of the untested carbon felt (heat treated at 400°C for 6 hours), CFs that have been tested at a cutoff voltage of 1.6 V and 1.8 V in 2 M H_2SO_4 electrolyte. For the untested felt (baseline), a reversible redox peak appeared at ~0.4 V, which is due to the carbonyl group. [3,4] In the potential region of 0.1 - 0.3 V, and 0.6 - 0.8 V, it behaves like a capacitor. However, for the 1.6 V felts, the redox peak was undetectable, and the capacitance seems to be smaller than the baseline. The oxidation peak at ~1.1 V and reduction peak at ~ 0.6 V are due to the $V^{4+/5+}$ redox couple, indicating that there is still residue vanadium electrolyte absorbed on the carbon felt electrode. The smaller capacitance for the 1.6 V felts might be due to the potential CF degradation during testing. Further insight into the individual electrodes, the 1.6 V (-) felt showed lower capacitance than the 1.6 V (+), which might indicate that the 1.6 V (-) felt experienced more severe degradation than the 1.6 V (+). For the 1.8 V (-) felt, large capacitance and two redox couples were observed, the carbonyl oxidation potential shifts to more positive than the untested felt, and a redox couple at 0.65 V appeared, which might be due to the

environment change of the carbonyl group. The significantly large capacitance might be associated with the over-reduction of the CF electrode. On the contrary, the 1.8 V (+) showed much lower capacitance, indicating that the 1.8 V (+) experienced more severe degradation than the 1.8 V (-) felt.

Figure S5 (b) shows the CV of $V^{4+/5+}$ on carbon felt electrodes. For the untested felt, the reaction is quasi-reversible, with a peak current ratio (Ipa/Ipc) of 2.52, and peak separation (Epa-Epc) of 0.248 V. On the tested felts, the oxidation peak shifts to more positive, and the reduction peak shifts to more negative potential, indicating slower reaction kinetics. This means that the tested felts are less active to the $V^{4+/5+}$ reaction, indicating carbon felt degradation. Table S1 compares the peak current, peak potential, Ipa/Ipc, and ΔEp obtained on these felts. It can be seen that the 1.6 V (-) showed more positive shift for the oxidation peak, and more negative shift for the reduction peak than the 1.6 V (+) felt. The peak current ratio and peak separation on the 1.6 V (-) is larger than that at 1.6 V (+) felt. This indicates that the 1.6 V (-) degraded more severely than the 1.6 V (+) felt, which is in agreement with that observed in Fig. S5. However, for the 1.8 V felts, the 1.8 V (+) felt degrades more severely than the 1.8 V (-), as demonstrated from the Ipa/Ipc ratio and ΔEp. The 1.8 V (+) felt showed irreversible $V^{4+/5+}$ behavior.

Table S1: Ipa, Ipc, Epa, Epc, Ipa/Ipc and ΔEp of carbon felts obtained from CV in 0.2 M $VOSO_4$-2M H_2SO_4

Felts	Ipa (mA mg^{-1})	Ipc (mA mg^{-1})	Epa (V)	Epc (V)	Ipa/Ipc	ΔEp (V)
Untested	1.391	0.551	0.962	0.714	2.52	0.248
1.6 V (+)	1.076	0.176	1.083	0.667	6.11	0.416
1.6 V (-)	0.800	0.049	1.123	0.582	16.33	0.541
1.8 V (+)	1.244	-*	1.166	-*	-*	-*
1.8 V (-)	1.592	0.286	1.100	0.627	5.57	0.473

* The Ipc, Epc, Ipa/Ipc, and Epa-Epc for the 1.8 V (+) were not listed in the table, since the reduction peak of V5 on 1.8 V (+) is very small, and cannot be extracted from the CV (Figure S5).

References

1 Q. Huang, C. Song, A. Crawford, Z. Jiang, A. Platt, K. Fatih, C. Bock, D. Jang and D. Reed, *RSC Adv.,* 2022, **12**, 32173.

2 D. Aaron, Z. Tang, A. B. Papandrew and T. A. Zawodzinski, *J. Appl. Electrochem.*, 2011, **41**, 1175.

3 A. K. Singh, M. Pahlevaninezhad, N. Yasri, E.P.L. Roberts, Degradation of carbon electrodes in the all-vanadium redox flow battery, ChemSusChem, 14 (2021) 2100–2111

4 T. J. Rabbow, M. Trampert, P. Pokorny, P. Binder, A.H. Whitehead, Variability within a single type of polyacrylonitrile—based graphite felt after thermal treatment. Part I: Chemical properties, Electrochim. Acta 173 (2015) 24 – 30
